# Association of Traditional and Emerging Anthropometric Indices With Subclinical Carotid Atherosclerosis in Young Healthy Adults: Insights From the Tehran Lipid and Glucose Study

**DOI:** 10.1002/edm2.70369

**Published:** 2026-10-22

**Authors:** Behnaz Abiri, Amirhossein Ramezani Ahmadi, Farhad Hosseinpanah, Pooneh Dehghan, Azra Ramezankhani, Maryam Mataji‐Amirroud, Nika Farsinajad, Fereidoun Azizi, Majid Valizadeh

**Affiliations:** ^1^ Obesity Research Center, Research Institute for Metabolic and Obesity Disorders, Research Institute for Endocrine Sciences Shahid Beheshti University of Medical Sciences Tehran Iran; ^2^ Isfahan Endocrine and Metabolism Research Center Isfahan University of Medical Sciences Isfahan Iran; ^3^ Department of Imaging, Taleghani Hospital Shahid Beheshti University of Medical Sciences Tehran Iran; ^4^ Prevention of Metabolic Disorders Research Center, Research Institute for Metabolic and Obesity Disorders, Research Institute for Endocrine Sciences Shahid Beheshti University of Medical Sciences Tehran Iran; ^5^ Research Center for Social Determinants of Health, Research Institute for Metabolic and Obesity Disorders, Research Institute for Endocrine Sciences Shahid Beheshti University of Medical Sciences Tehran Iran; ^6^ Endocrine Research Center, Research Institute for Endocrine Disorders, Research Institute for Endocrine Sciences Shahid Beheshti University of Medical Science Tehran Iran

**Keywords:** a body shape index, anthropometric indices, body roundness index, cardiovascular risk, carotid intima‐media thickness, waist‐to‐height ratio

## Abstract

**Background:**

Obesity is a major risk factor for cardiovascular disease, but traditional measures like BMI and waist circumference have limitations. New indices, BRI and ABSI, may better reflect fat distribution and cardiovascular risk. This study examined their association with carotid intima‐media thickness (cIMT) in young healthy adults.

**Methods:**

In this cross‐sectional study within the Tehran Lipid and Glucose Study (TLGS) framework, 572 adults aged 20–40 years underwent anthropometric assessments, biochemical tests and carotid ultrasound for cIMT measurement. Anthropometric indices including BMI, WC, waist‐to‐height ratio (WHtR), ABSI and BRI were calculated. Associations between these indices and cIMT were analysed using correlation and multivariable linear regression models adjusting for demographic, metabolic and lifestyle factors. Receiver operating characteristic (ROC) curve analyses assessed the predictive ability of the indices for elevated cIMT.

**Result:**

BRI and WHtR were nearly collinear (*r* = 0.997) and showed modest positive correlations with cIMT in unadjusted analyses (*r* = 0.103 for both), but these associations weakened after adjustment for cardiometabolic risk factors. WC was the only independent predictor of cIMT in partially adjusted models, though this significance disappeared in fully adjusted analyses. ABSI exhibited a significant but mechanistically unexpected inverse association with cIMT. ROC analyses indicated limited discriminative power for all anthropometric measures in predicting high cIMT, with BRI and WHtR having the highest but still modest area under the curve (AUC).

**Conclusion:**

In young healthy adults, BRI and WHtR showed modest associations with subclinical carotid atherosclerosis, which weakened after adjustment. Waist circumference was an inconsistent predictor, and ABSI demonstrated an inverse relationship with cIMT. Further longitudinal studies are needed to confirm their clinical utility.

## Graphical abstract

No anthropometric index—traditional (BMI, WC, WHtR) or emerging (BRI, ABSI)—significantly discriminated elevated carotid intima‐media thickness in 572 young healthy adults. All AUCs were close to chance, and associations attenuated after full adjustment. Findings do not support clinical use of these indices in this population.

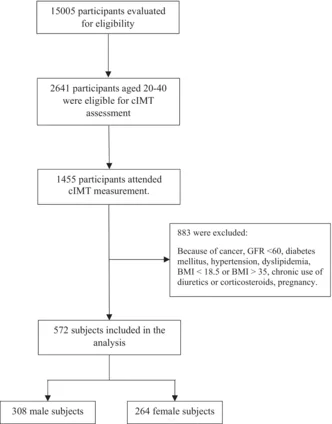

## 1 Introduction

Obesity independently elevates the risk of cardiovascular disease and death [1]. Numerous studies have sought to establish objective and consistent obesity indicators that can accurately quantify fat accumulation and clarify its relationship with cardiovascular events [1].

Body mass index (BMI), defined as weight in kilograms divided by height in meters squared (kg/m^2^), is the most commonly used anthropometric indicator for classifying overweight and obesity in clinical settings and epidemiological research [2, 3, 4], and it is endorsed by the World Health Organization (WHO) [5]. However, this anthropometric index cannot differentiate between fat and lean body mass, nor can it specify the distribution of fat in central versus peripheral regions [6]. These limitations are well documented: Nuttall [4] showed that BMI poorly reflects percent body fat and fat distribution, while Pischon et al. [2] in 359,387 EPIC participants, demonstrated that both general (BMI) and abdominal (WC, WHR) adiposity independently predicted mortality, with WC and WHR remaining significant even after adjustment for BMI. Consequently, waist circumference (WC) and waist‐to‐hip ratio (WHR) have been introduced as measures of central obesity due to their ability to reflect fat distribution [6].

WC is widely used but its measurement site varies across guidelines—the WHO and IDF use the midpoint between the lowest rib and the iliac crest, whereas the NIH uses the superior border of the iliac crest—potentially affecting comparability across studies. A significant limitation of WC is that it does not take into account an individual's height and weight [7], which can lead to an overestimation or underestimation of obesity in tall or short persons [8]. To address this, the waist‐to‐height ratio (WHtR)—WC (cm) divided by height (cm)—was proposed. Meta‐analyses show WHtR outperforms both BMI and WC for screening cardiometabolic risk [8] and predicts risk even in non‐obese populations. Since WHR provides no significant benefit compared to WC for obesity evaluation [6], it is not commonly used in clinical practice.

Consequently, additional indices have been developed to address the limitations of traditional obesity markers. The Body Roundness Index (BRI), introduced by Thomas et al. [9], models the body as an ellipse (height as major axis, WC as minor axis), with its eccentricity transformed into the BRI scale. Critically, BRI is a one‐to‐one nonlinear transformation of WHtR, so the two rank individuals identically. In restricted BMI ranges (18.5–35 kg/m^2^), they perform near‐identically, with empirical correlations exceeding *r* = 0.99. Similarly, Krakauer et al. [10] introduced A Body Shape Index (ABSI), which adjusts WC for height and weight to provide a more refined assessment of body shape. Studies show that ABSI is more closely associated with abdominal fat accumulation [10], the risk of early death [10], and diabetes development [11] compared to BMI. However, subsequent studies have raised substantial questions about ABSI's clinical utility [12, 13].

Carotid intima‐media thickness (cIMT), measured by B‐mode ultrasound, is a structural marker of early atherosclerotic burden, distinct from functional arterial stiffness measures such as pulse wave velocity (PWV). It independently predicts future cardiovascular events [14] and was selected as the primary outcome because it is a validated, non‐invasive measure of subclinical atherosclerosis with well‐established normative data in young adults. Young adults (20–40 years) were selected because atherosclerosis begins early in life, subclinical disease is common in this group, and current risk algorithms inadequately cover individuals under 40 years.

However, findings regarding the association between these emerging obesity indices and cardiovascular disease (CVD) risk are inconsistent [15, 16], and there is a scarcity of research on the prognostic value of these markers in individuals with arterial health [17]. Cardiovascular disease is the foremost cause of death from non‐communicable diseases worldwide and continues to pose a significant global health challenge [17]. Therefore, early identification and management of CVD risk factors are crucial to prevent disease progression and reduce adverse health outcomes. Arterial stiffness is an independent predictor of CVD risk [17].

Accordingly, as traditional obesity measures like BMI and WC have limitations in reflecting fat distribution and predicting early vascular changes, emerging indices such as BRI and ABSI may offer better insights, but their relationship with subclinical carotid atherosclerosis in young adults remains unclear. Moreover, WHtR—despite its established performance in predicting cardiometabolic risk—has been less frequently evaluated against cIMT in young healthy populations. This study aims to assess the association between traditional and emerging anthropometric indices—including BMI, WC, WHtR, BRI and ABSI—and cIMT in young healthy adults.

## 2 Methods

### 2.1 Study Population

This cross‐sectional study was carried out within the framework of the Tehran Lipid and Glucose Study (TLGS). The TLGS is a community‐based cohort study aimed at identifying risk factors and outcomes of non‐communicable diseases [18, 19]. The researchers employed a multistage random sampling technique to select participants aged over 3 years for the study. The participant selection was conducted in Tehran's 13th district. They underwent follow‐up assessments every three years to update measurements of anthropometric examinations, demographic details, biochemical profiles, clinical features and lifestyle factors. In the initial phase, a baseline cross‐sectional survey was carried out between 1999 and 2001. Subsequently, a prospective follow‐up study was conducted from 2002 to 2005 (Phase II). Phases 3–5 were conducted every three years until 2016, followed by Phase VI from 2016 to 2019 and Phase VII from 2019 to 2021 as subsequent stages of the study.

Among all participants who took part in the initial phase and completed every follow‐up through the seventh phase, 2641 individuals qualified for the cIMT assessment during Phase VI (2016–2019) of the TLGS. Among these, 1455 participants attended the cIMT measurement, and from this group, 572 individuals aged 20–40 met the inclusion criteria for our study. The cIMT measurements were carried out from February 2017 through October 2019. The exclusion criteria of this study included people with cancer (*n* = 3), GFR < 60 (*n* = 5), chronic use of diuretics (*n* = 10), chronic use of corticosteroids (*n* = 34), individuals with BMI < 18.5 or BMI > 35 kg/m^2^ (*n* = 112), those with diabetes mellitus (*n* = 17) or hypertension (*n* = 43) or dyslipidemia (*n* = 647), and participants who were pregnant (*n* = 12).

Participants with BMI < 18.5 or > 35 kg/m^2^ were excluded to reduce physiological heterogeneity. Underweight individuals represent a distinct group in whom low BMI may reflect malnutrition or illness rather than leanness, and severely obese individuals are likely to have distinct pathophysiological mechanisms and comorbidities that could confound the association. This BMI range is consistent with other studies of early subclinical atherosclerosis in young populations.

Finally, a total of 572 subjects were included in our study, comprising 308 males and 264 females (Figure 1).

**FIGURE 1:**
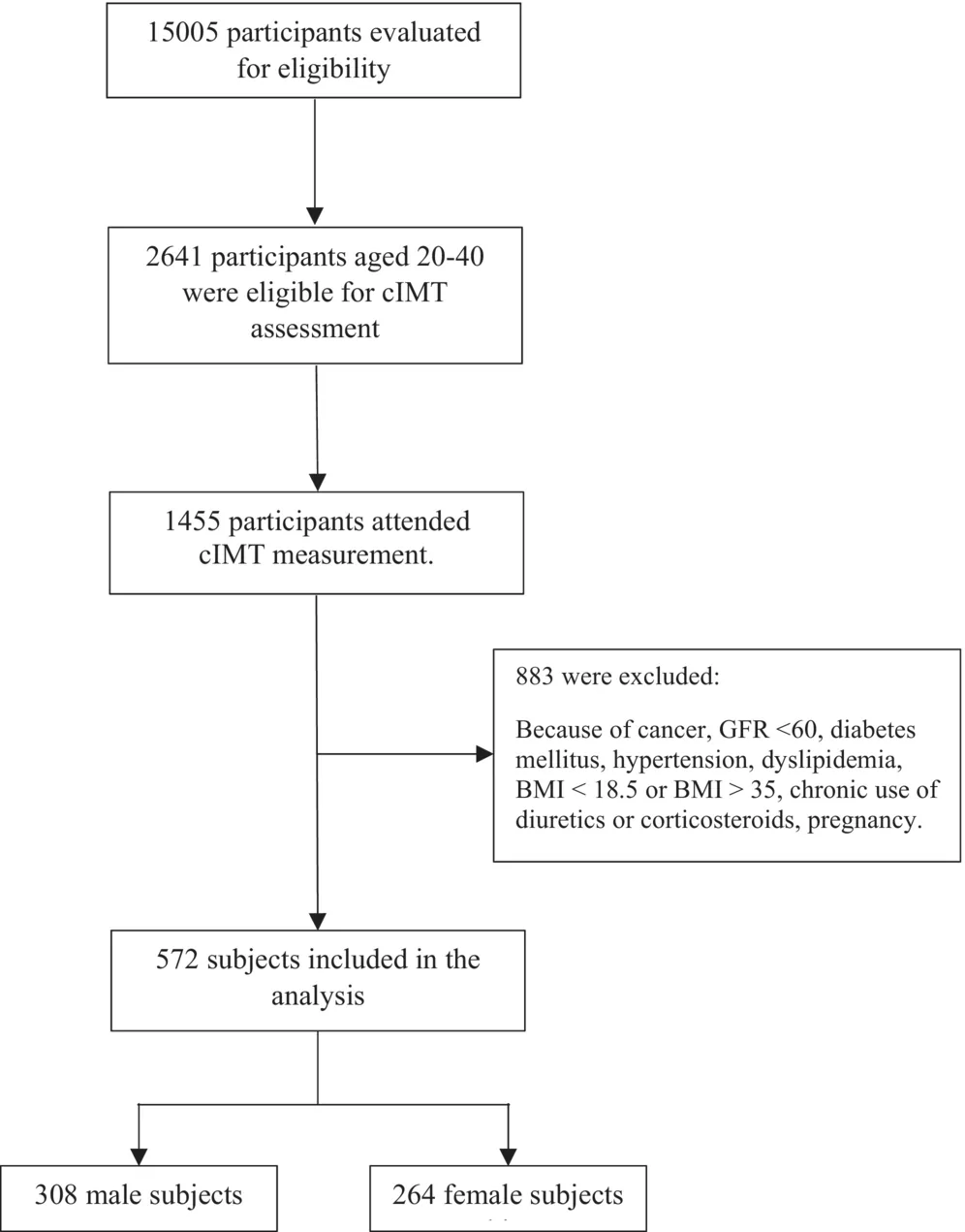
The flowchart of recruitment.

This study was approved by the ethics committee of Research Institute for Endocrine Sciences (RIES) of Shahid Beheshti University of Medical Sciences (code: IR.SBMU.ENDOCRINE.REC.1403.041). All ethical principles outlined in the Declaration of Helsinki were strictly adhered to, and each participant provided written informed consent before enrollment.

### 2.2 Anthropometric Assessment, Laboratory Measurement, Assessment of the cIMT

#### 2.2.1 Anthropometric Measurements

Qualified healthcare professionals conducted anthropometric measurements following established standard protocols to ensure accuracy and consistency [18, 19]. The participant's weight was measured on a digital scale while wearing minimal clothing and without footwear. The scale provided measurements to the nearest 0.1 kg. (Seca707; range 0.1–150 kg, Hanover, MD, USA). The participant's height was measured using a tape measure stadiometer while standing against a wall, with shoulders naturally aligned. Waist circumference was measured using a non‐stretch tape at the narrowest point between the lowest rib and the iliac crest, with the participant standing relaxed, without applying pressure to the tape, and exhaling at the end of the measurement. This measurement site is consistent with the WHO protocol. Alternative protocols, including the NIH protocol (midpoint between the lowest rib and the iliac crest) and the IDF protocol (just above the iliac crest), may produce systematically different WC values, particularly in individuals with android fat distribution. Because WC is a component of WHtR, WHR, BRI and ABSI, protocol‐related differences in WC may propagate through all derived indices. Therefore, the WC values and derived indices in this study may not be directly comparable with those from studies using different measurement sites. Hip circumference was measured over light clothing at the widest part of the buttocks, and height, WC, and HC were recorded to the nearest 0.1 cm. Although the TLGS protocol specifies measurement over light clothing, this may introduce a small degree of measurement error that could affect the precision of WHR estimates. However, all measurements were performed by trained healthcare professionals following standardized protocols, and the potential for systematic error is expected to be minimal.

Body mass index (BMI) was calculated by dividing the participant's weight in kilograms by the square of their height in meters. Waist‐to‐height ratio (WHtR) was calculated by dividing WC (cm) by height (cm), while waist‐to‐hip ratio (WHR) was computed by dividing WC (cm) by HC (cm).

ABSI was computed using the following formula [9]:

$$\text{ABSI}=\mathrm{WC}/\left({\mathrm{BMI}}^{\left(2/3\right)}\times {\text{Height}}^{\left(1/2\right)}\right)$$

BRI was calculated using the formula [10]:

$$\mathrm{BRI}=364.2–365.5\times \surd \left(1-\left({\left(\mathrm{WC}/\left(\text{2π}\right)\right)}^{2}/{\left(0.5\times \text{height}\right)}^{2}\right)\right)$$

### 2.3 Measurement of Metabolic Indices and cIMT

A trained medical professional measured each participant's systolic and diastolic blood pressure twice while the participant was seated, after resting for 15 min. The initial measurement was taken using a mercury sphygmomanometer to establish the peak inflation pressure. The participants' blood pressure was calculated by averaging the two recorded measurements.

In accordance with standard protocol, blood samples were collected from participants between 7 and 9 a.m., following a fasting period of 12–14 h. Following collection, samples were centrifuged for 30–45 min to ensure proper separation. The TLGS research laboratory conducted same‐day analyses of the serum and centrifuged blood samples collected from participants, measuring lipid concentrations and fasting plasma glucose (FPG) levels. The laboratory used kits available in the Iranian market, supplied by Pars Azmoun Company in Tehran, Iran, which were calibrated for compatibility with the Selectra 2 automatic analyser.

Plasma glucose concentration was measured using the enzyme colorimetric method based on glucose oxidase. The FPG assay exhibited a coefficient of variation (CV) of 2.2%. To measure triglycerides (TG), an enzymatic colorimetric method utilizing glycerol phosphate oxidase was employed. The CV were 0.6% for inter‐assay and 1.6% for intra‐assay measurements. Total cholesterol (TC) was measured using an enzymatic colorimetric method involving cholesterol esterase and cholesterol oxidase enzymes.

High‐density lipoprotein cholesterol (HDL‐C) levels were determined by precipitating apolipoprotein B‐containing lipoproteins using phosphotungstic acid. The CVs were determined to be 0.5% for TC and 2% for HDL‐C. When the TG concentration was under 400 mg/dL, the Friedewald formula was used to calculate the LDL‐C level based on the serum concentrations of TC, TG and HDL‐C [20].

During the study, participants were examined using a Samsung Medison SonoAce R3 ultrasound system equipped with a linear transducer operating at a frequency range of 7.5–10 MHz. Two radiologists, blinded to participant information, conducted the ultrasound examinations with the participants lying supine, their necks extended and slightly rotated away from the examiner. Measurements were conducted on both the left and right carotid arteries; however, the left common carotid artery (LCCA) was selected for analysis based on precedent established in previously published studies [21, 22]. The reported cIMT values represent the mean of three measurements taken at distinct sites on the distal LCCA. The right common carotid artery was scanned for completeness and to assess for plaques, but these measurements were not included in the analysis.

An initial scan of the carotid artery was performed in the transverse plane along its path to assess the participant's vascular anatomy and identify any atherosclerotic plaques. The site of greatest wall thickening was identified on either the proximal or distal wall. Subsequently, a longitudinal scan was performed from multiple angles to further evaluate the area.

We selected arterial segments that were free from plaques and met established criteria for optimal B‐mode ultrasound imaging. The criteria required clear visualization of the arterial interfaces on the distal wall, with the lumen appearing completely anechoic, optimal depth achieved by centering the arterial lumen within the image, and the focal zone adjusted precisely at the level of the lumen. This method enabled us to acquire high‐resolution grayscale images of the carotid artery, which were then stored for subsequent analysis. The scanning depth was modified to position the arterial lumen at the center of the image, with the focal zone adjusted accordingly to enhance image clarity.

During the cIMT assessment, a hypoechoic band was observed representing the area between the echogenic intimal layer and other arterial wall layers. The distance between the leading edges of the first and second echogenic lines on the peripheral walls of the distal common carotid artery was measured at three distinct sites on both sides. The final result was calculated as the average of these measurements. Electrocardiography (ECG) was not performed during the ultrasound scan, so the precise phase of the cardiac cycle at which the measurements were taken could not be identified.

The cIMT of the carotid bulb and internal carotid artery was measured on both sides in participants who fulfilled the optimal imaging and technique criteria, although measurements were taken sporadically.

To assess reliability and agreement, two radiologists independently measured the cIMT in 30 participants (66.7% female), whose mean age and BMI were 41.7 ± 10.7 years and 24.4 ± 5.5 kg/m^2^, respectively. The intraclass correlation coefficient (ICC) was employed to assess the level of agreement between the two radiologists in measuring the cIMT parameters. ICC estimates and their 95% confidence intervals were calculated using SPSS version 20, based on a two‐way mixed effects model. The ICC was 0.79, with a 95% confidence interval ranging from 0.55 to 0.90. ICC values vary between 0 and 1, where values from 0.75 to 0.9 are indicative of good reliability [23]. Additionally, the difference in cIMT measurements between the two radiologists showed a mean (±SD) of 0.08 (±0.12) mm. The reliability subsample was older than the analytic sample but had a comparable BMI distribution. Because cIMT measurement error is influenced by image quality, which tends to be better in younger individuals with less atherosclerotic burden, the ICC of 0.79 may represent a conservative estimate of reliability in the younger analytic sample.

### 2.4 Physical Activity Assessment

Physical activity was evaluated using the modifiable activity questionnaire (MAQ), a validated instrument that assesses the frequency and duration of physical activities across various domains, such as walking, moderate‐intensity and vigorous‐intensity activities, over the previous 7 days [24]. Participants reported the duration of each activity, which was then used to calculate their total physical activity level in Metabolic Equivalent of Task (MET) minutes per week. Based on their total MET‐minutes per week, participants were categorized into three groups: low physical activity (< 600 MET‐minutes/week), moderate physical activity (600–2999 MET‐minutes/week), and high physical activity (≥ 3000 MET‐minutes/week).

### 2.5 Statistical Analysis

Statistical analyses were performed using SPSS version 20.0, with a significance level of *p* < 0.05. Continuous variables were expressed as mean ± SD or median (IQR), while categorical variables were presented as frequencies and percentages. Group differences across ABSI and BRI quartiles were assessed using one‐way ANOVA or Kruskal‐Wallis tests for continuous variables and Chi‐square tests for categorical variables. Correlations between ABSI, BRI and cIMT were analysed using Pearson's or Spearman's correlation coefficients. Multiple linear regression models adjusted for age, sex, BMI, blood pressure, lipid profile, fasting plasma glucose and physical activity evaluated the independent associations of ABSI and BRI with cIMT. Sub‐group analyses included stratification by sex for the ROC analyses. Regression models were not stratified by sex due to limited statistical power within strata. The intraclass correlation coefficient (ICC) assessed cIMT measurement reliability, and data visualization included scatter plots and box plots to illustrate key findings.

This study is a secondary analysis nested within the TLGS cohort. No a priori sample size calculation was performed; the sample size was determined by the number of eligible TLGS participants who attended cIMT measurement during Phase VI and met the inclusion criteria. All eligible participants were included (*n* = 572). With 89 participants in the high cIMT group, the study's statistical power to detect modest differences in AUC between anthropometric indices is likely low.

## 3 Results

### 3.1 General and Clinical Characteristics

A total of 572 young adults participated in the study (53.8% male, 46.2% female). Table 1 presents the general and clinical characteristics of the study population overall and categorized into low and high cIMT groups based on their cIMT percentile (< 75th percentile or > 75th percentile of cIMT). Totally 483 participants were under 75th percentile and 89 of them were above the 75th percentile. The mean age of participants was 28.55 ± 4.07 years. No significant difference was observed in age, education, marital status, smoking status, physical activity level, SBP, DBP, TG, TC, LDL‐c, HDL‐c and FBS between the two categories of cIMT. However, the proportion of males was significantly higher in the < 75th percentile group (57.3%) compared to the > 75th percentile group (34.8%) (*p* < 0.0001). Also, the high cIMT group had a lower GFR (*p* = 0.007) compared to the low cIMT group. In addition, the bivariate correlation (Table 2) indicated cIMT (as a quantitative variable) has a significant negative correlation with GFR (*r* = −0.107) and physical activity (*r* = −0.087).

**TABLE 1:** Demographic and clinical data of the study population based on cIMT values lower and greater than 75th percentile.

Variables	Overall population (*n* = 572)	IMT < 75th percentile (*n* = 483)	IMT > 75th percentile (*n* = 89)	*p*
Age, years	28.55 ± 4.07	28.46 ± 4.06	29.03 ± 4.13	0.224
Sex				
Male	308 (53.8)	277 (57.3)	31 (34.8)	**< 0.0001**
Female	264 (46.2)	206 (42.7)	58 (65.2)	
Education				
< 12 years	342 (60)	283 (58.7)	59 (67)	0.177
≥ 12 years	228 (40)	199 (41.3)	29 (33)	
Marital status				
Single	327 (57.3)	280 (58)	47 (53.4)	0.419
Married	239 (41.9)	198 (41)	41 (46.6)	
Widow	5 (0.9)	5 (1)	0	
Height, cm	169.47 ± 10.07	169.82 ± 10.09	167.54 ± 9.80	0.054
Weight, kg	70.75 ± 13.85	70.80 ± 13.68	70.52 ± 14.86	0.868
BMI, kg/m^2^	24.51 ± 3.52	24.42 ± 3.45	24.99 ± 3.89	0.170
WC, cm	84.79 ± 9.73	84.66 ± 9.67	85.49 ± 10.68	0.474
WHtR	0.50 ± 0.05	0.49 ± 0.05	0.51 ± 0.05	0.061
HC, cm	97.73 ± 7.63	97.40 ± 7.64	99.55 ± 7.34	**0.017**
WHR	0.86 ± 0.07	0.86 ± 0.07	0.85 ± 0.07	0.209
BRI	1.05 ± 0.51	1.03 ± 0.49	1.15 ± 0.59	0.051
ABSI	0.13 ± 0.01	0.13 ± 0.008	0.12 ± 0.008	**< 0.0001**
GFR	83.91 ± 10.84	84.45 ± 11.10	80.98 ± 8.83	**0.007**
SBP, mmHg	105.42 ± 10.90	105.27 ± 10.93	106.23 ± 10.72	0.455
DBP, mmHg	70.80 ± 8.05	70.81 ± 8.24	70.72 ± 6.92	0.925
TG, mg/dl	80.14 ± 26.23	80.64 ± 26.68	77.44 ± 23.57	0.304
Cholesterol, mg/dl	161.12 ± 21.68	161.15 ± 21.92	160.96 ± 20.47	0.942
LDL‐c, mg/dl	91.46 ± 19.50	91.48 ± 19.57	91.38 ± 19.24	0.966
HDL‐c, mg/dl	53.62 ± 8.76	53.54 ± 9.00	54.09 ± 7.37	0.595
FBS, mg/dl	87.74 ± 7.33	87.71 ± 7.41	87.90 ± 6.92	0.831
Physical activity, Mets/week	2515 ± 3695	2579 ± 3830	2157 ± 2808	0.341
Yes	104 (19.1)	95 (20.5)	9 (11)	0.061
No	441 (80.9)	368 (79.5)	73 (89)	

*Note:* The bold values indicate statistical significance (*p* < 0.05).

Abbreviations: ABSI, a body shape index; BMI, body mass index; BRI, body roundness index; cIMT, carotid intima‐media thickness; DBP, diastolic blood pressure; FBS, fasting blood sugar; GFR, glomerular filtration rate; HC, hip circumference; HDL‐c, high‐density lipoprotein cholesterol; LDL‐c, low‐density lipoprotein cholesterol; SBP, systolic blood pressure; TG, triglycerides; WC, waist circumference; WHR, waist‐to‐hip ratio; WHtR, waist‐to‐height ratio.

**TABLE 2:** The bivariate correlation between cIMT and anthropometric parameters and other variables in the study^a^.

	cIMT	BMI	WC	WHtR	BRI	ABSI
Age, years	0.077	0.034	0.038	0.128^b^	0.119^b^	−0.035
Education	−0.060	0.064	0.067	0.013	0.013	−0.035
Height, cm	−0.106^b^	0.086^c^	0.403^b^	−0.123^b^	−0.119^b^	0.805^b^
Weight, kg	−0.018	0.789^b^	0.867^b^	0.559^b^	0.560^b^	0.532^b^
BMI, kg/m^2^	0.061	—	0.834^b^	0.856^b^	0.857^b^	0.051
WC, cm	0.042	0.834^b^	—	0.857^b^	0.855^b^	0.568^b^
WHtR	0.103^c^	0.856^b^	0.857^b^	—	0.997^b^	0.165^b^
HC, cm	0.092^c^	0.727^b^	0.601^b^	0.619^b^	0.613^b^	0.030
WHR	−0.030	0.447^b^	0.771^b^	0.569^b^	0.569^b^	0.714^b^
BRI	0.103^b^	0.857^b^	0.855^b^	0.997^b^	—	0.165^b^
ABSI	−0.049	0.051	0.568^b^	0.165^b^	0.162^b^	—
GFR	−0.107^b^	0.036	0.067	0.023	0.026	0.079
SBP, mmHg	−0.004	0.291^b^	0.329^b^	0.155^b^	0.159^b^	0.252^b^
DBP, mmHg	0.016	0.157^b^	0.238^b^	0.128^b^	0.131^b^	0.229^b^
TG, mg/dl	−0.035	0.271^b^	0.361^b^	0.253^b^	0.247^b^	0.270^b^
Cholesterol, mg/dl	0.016	0.146^b^	0.117^b^	0.177^b^	0.172^b^	−0.036
LDL‐c, mg/dl	0.018	0.195^b^	0.199^b^	0.194^b^	0.191^b^	0.066
HDL‐c, mg/dl	0.021	−0.235^b^	−0.368^b^	−0.145^b^	−0.146^b^	−0.398^b^
FBS, mg/dl	−0.003	0.097^c^	0.158^b^	0.081	0.080	0.169^b^
Physical activity	−0.087^c^	0.007	0.038	−0.041	−0.041	0.101^c^
Smoking	−0.080	−0.014	0.098^c^	−0.074	−0.074	0.249^b^

^a^ Correlation coefficients are calculated using Pearson for quantitative variables and Spearman for qualitative variables.

^b^ Correlation is significant at the 0.01 level.

^c^ Correlation is significant at the 0.05 level.

### 3.2 The Association Between Anthropometric Parameters and cIMT

The independent sample *t*‐test (Table 1) found no significant differences in height, weight, BMI, WC, WHtR or WC/HC ratio between the two cIMT groups. However, the BRI was marginally higher in the > 75th percentile group (1.15 ± 0.59 vs. 1.03 ± 0.49, *p* = 0.051). Interestingly, ABSI was significantly lower in individuals with higher cIMT (*p* < 0.0001). HC was significantly greater in the high cIMT group (*p* = 0.017).

Table 2 presents the correlation of anthropometric parameters, including BMI, WC, WHtR, BRI and ABSI with studied variables. Bivariate correlation showed a significant positive correlation between cIMT and BRI (*r* = 0.103, *p* < 0.01), WHtR (*r* = 0.103, *p* < 0.05), and HC (*r* = 0.092, *p* < 0.05), but not with BMI (*r* = 0.061, *p* > 0.05), WC (*r* = 0.042, *p* > 0.05), and ABSI (*r* = −0.049, *p* > 0.05).

In unadjusted multivariable linear regression models (Table 3), WHtR (*B* = 0.15, *p* = 0.029) and BRI (*B* = 0.01, *p* = 0.028) were associated with cIMT. After adjusting for the effect of sex and age, WC (*B* = 0.001, *p* = 0.022) and BRI (*B* = 0.01, *p* = 0.048) were independently associated with cIMT. In model 3, after adjusting for the effect of age, sex, education level, smoking status and physical activity level, only the association between WC and cIMT was statistically significant (*B* = 0.001, *p* = 0.022). Moreover, in model 4, none of anthropometric parameters were associated with cIMT after adjusting for the effect of SBP, DBP, TG, TC, HDL‐c, LDL‐c, FBS and GFR other than variables in previous models.

**TABLE 3:** Multivariate linear regression for association between cIMT and anthropometric parameters.

	Model 1^a^			Model 2^b^			Model 3^c^			Model 4^d^		
	*B* ^e^	SE	*p*	*B* ^e^	SE	*p*	*B* ^e^	SE	*p*	*B* ^e^	SE	*p*
BMI	0.001	0.001	0.209	0.002	0.001	0.076	0.002	0.001	0.084	0.001	0.001	0.258
WC	0.0001	0.0004	0.416	0.001	0.0004	**0.022**	0.001	0.0004	**0.025**	0.001	0.0005	0.088
WHtR	0.15	0.07	**0.029**	0.14	0.07	0.052	0.13	0.07	0.063	0.11	0.07	0.151
BRI	0.01	0.007	**0.028**	0.01	0.007	**0.048**	0.01	0.007	0.059	0.01	0.008	0.145
ABSI	−0.41	0.35	0.239	0.65	0.45	0.152	0.66	0.45	0.146	0.57	0.45	0.210

*Note:* The bold values indicate statistical significance (*p* < 0.05).

^a^ The value of cIMT was entered as the response variable and anthropometric parameters were entered as predictor variables in the unadjusted model.

^b^ The effects of age and sex were adjusted.

^c^ The effects of age, sex, education level, smoking status and physical activity level were adjusted.

^d^ The effects of age, sex, education level, smoking status, physical activity level, SBP, DBP, TG, TC, HDL‐c, LDL‐c, FBS and GFR were adjusted.

^e^ Values are regression coefficients (*B*), representing the change in cIMT (mm) per one‐unit increase in each anthropometric index.

The ABSI coefficient reversed sign across models, from *B* = −0.41 (*p* = 0.239) in the unadjusted model to *B* = +0.65 (*p* = 0.152) after adjustment for age and sex, and remained positive in Models 3 (*B* = +0.66, *p* = 0.146) and 4 (*B* = +0.57, *p* = 0.210). This sign reversal reflects suppression by sex.

### 3.3 Predictive Ability (ROC Analysis)

Figure 2 shows receiver operating characteristic (ROC) curve for different anthropometric parameters in predicting cIMT. Also, the AUC of each parameter is presented in Table 4. ROC curve analysis revealed no significant discriminative ability for any anthropometric index in predicting elevated cIMT. The AUCs ranged from 0.457 to 0.549 across all indices and all analyses (overall population and sex subgroups), and none was significantly different from 0.5 (*p* > 0.05 for all comparisons, Table 4). WHtR and BRI yielded the numerically highest AUC (0.549 ± 0.035), but this value was statistically indistinguishable from chance. The AUC for ABSI was numerically the lowest (0.457 ± 0.033, *p* = 0.202), also indicating no discriminative ability. In sex‐stratified analyses, BMI had the numerically highest AUC in males (0.556 ± 0.060) and WC in females (0.570 ± 0.043), but neither reached statistical significance. These findings indicate that none of the anthropometric indices examined demonstrated meaningful discriminative ability for elevated cIMT in this population.

**FIGURE 2:**
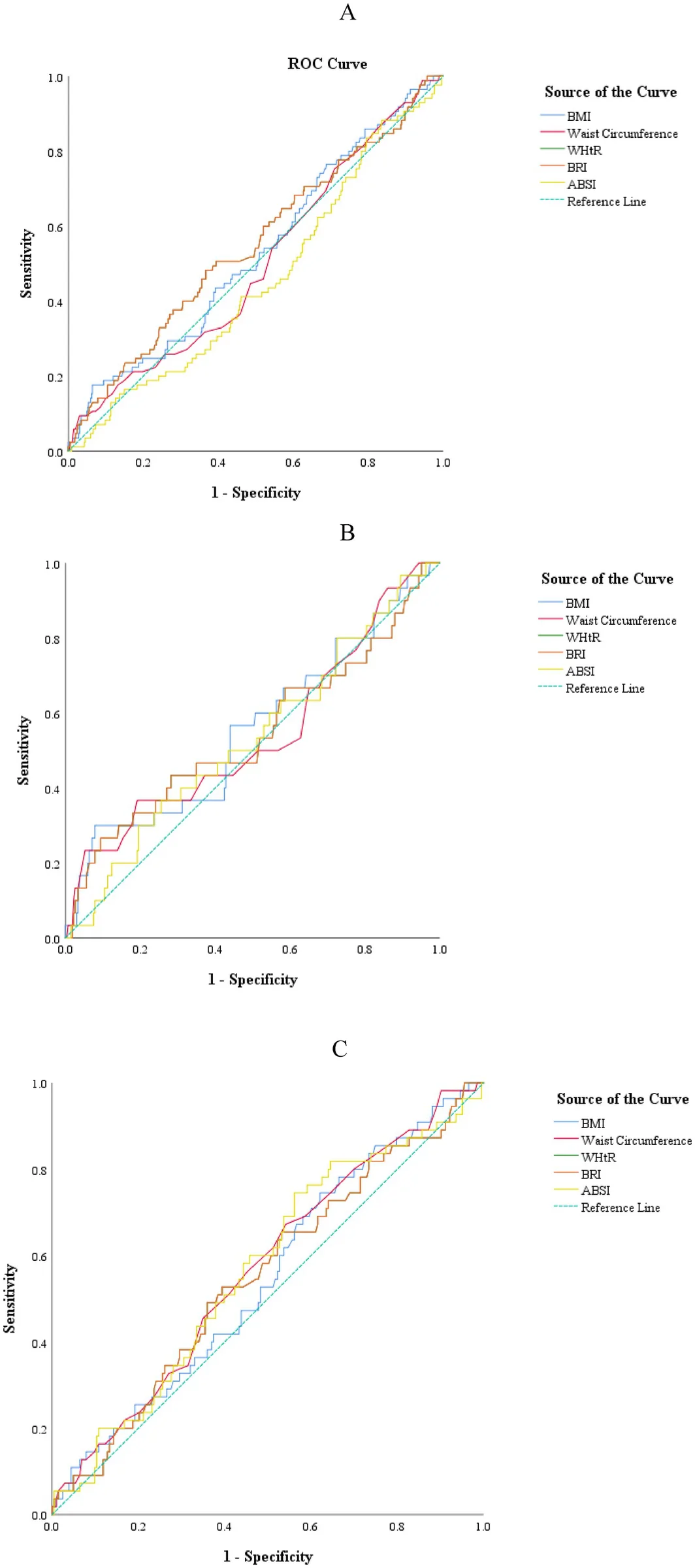
Receiver operating characteristic (ROC) curves for anthropometric indices (BMI, WC, WHtR, BRI and ABSI) in predicting carotid intima‐media thickness (cIMT) above the 75th percentile among young adults: (A) overall population, (B) males and (C) females.

**TABLE 4:** Area under curve values for anthropometric parameters in predicting cIMT > 75th percentile in overall population and divided based on gender.

	Overall		Male		Female	
	AUC ± SE	*p*	AUC ± SE	*p*	AUC ± SE	*p*
BMI	0.530 ± 0.034	0.378	0.556 ± 0.060	0.311	0.548 ± 0.043	0.272
WC	0.501 ± 0.034	0.967	0.540 ± 0.061	0.468	0.570 ± 0.043	0.114
WHtR	0.549 ± 0.035	0.149	0.543 ± 0.063	0.438	0.551 ± 0.044	0.242
BRI	0.549 ± 0.035	0.149	0.543 ± 0.063	0.438	0.551 ± 0.044	0.242
ABSI	0.457 ± 0.033	0.202	0.533 ± 0.056	0.558	0.567 ± 0.043	0.126

## 4 Discussion

The present study investigated the association between anthropometric indices and cIMT in young adults. BRI and WHtR showed modest correlations with cIMT in unadjusted analyses that attenuated after adjustment; WC was the sole independent predictor in partially adjusted models but lost significance upon full adjustment; ABSI showed an inverse relationship; and ROC analyses demonstrated no significant discriminative ability for any index. The strongest univariate finding was a sex difference in cIMT group membership—males predominated in the low cIMT group (57.3% vs. 34.8%, *p* < 0.0001), directionally opposite to older adults—possibly reflecting greater lean mass in males, counteracting factors to oestrogen's vasoprotective effects in females, or selective participation by sex. Future studies should consider sex as a potential effect modifier rather than merely a confounder.

It is important to distinguish cIMT from PWV: cIMT reflects structural atherosclerotic burden, whereas PWV reflects functional arterial stiffness, and findings from PWV studies cannot be directly extrapolated to cIMT. Moreover, the null ROC findings should be interpreted cautiously because the 75th percentile threshold was internally derived and not clinically validated; published normative data indicate that cIMT values around 0.5 mm are normal in young adults (mean 0.57–0.59 mm at ages 24–39). Thus, the ‘high cIMT’ group may not represent genuinely elevated cardiovascular risk, and the null findings likely reflect an inability to discriminate within the normal range.

Previous studies identify obesity measurements as risk factors for arterial stiffness, but findings remain inconsistent [25, 26, 27]. For instance, a study from Japan found that ABSI had the highest AUC (0.73) among several anthropometric indices for elevated cardio‐ankle vascular index (CAVI) [28]. This apparent advantage, however, should be interpreted cautiously, because CAVI reflects functional arterial stiffness rather than structural atherosclerotic burden, and therefore cannot be directly extrapolated to cIMT. WHO recommends BMI and WC [29, 30], yet BMI does not differentiate muscle from fat [17] or capture fat distribution [31], and WC does not account for height, leading to underestimation in shorter and overestimation in taller individuals [7, 8]. Accordingly, ABSI and BRI were proposed [9, 10]. BRI more accurately predicts body fat and visceral adipose tissue than BMI and WC [9], though evidence for its predictive ability is mixed [15, 32, 33, 34, 35]. ABSI, designed as a WC‐based index independent of BMI [10], shows inconsistent associations with cardiovascular risk factors [11, 15, 36, 37, 38]; in 4627 Dutch adults it did not detect cardiovascular events [15], and Fujita et al. [39] found it less effective than BMI or WC in 48,953 Japanese adults. Differences may stem from ethnic, racial, and sex‐based variations in body height and WC.

WHtR has been identified as the best predictor of coronary heart disease risk in women [40, 41] and is more efficient than BMI and WC for screening metabolic risks [8]. Consistent with our results, WHtR predicts cardiovascular risk and metabolic syndrome even in non‐obese populations [8, 42], highlighting its value as a diagnostic tool for central obesity.

The near‐perfect collinearity between BRI and WHtR (*r* = 0.997) is the most important analytical feature of this dataset. BRI is a one‐to‐one nonlinear transformation of WHtR—derived from an elliptical model using height and WC—and the restricted BMI range (18.5–35 kg/m^2^) reinforces this equivalence. Krakauer and Krakauer [43], using NHANES III data, confirmed that BRI is mathematically equivalent to WC/height and that both WHtR and BRI correlate 0.91 with BMI (0.857 in our dataset), indicating that neither is independent of BMI. Three implications follow: (i) BRI and WHtR are the same construct, so the study compares four constructs, not five; (ii) BRI offers no ‘novel’ predictive value beyond WHtR, as confirmed by identical AUC values (0.549 for both); and (iii) WHtR is simpler, more intuitive, and has a universal cutoff (0.50), whereas BRI adds complexity without benefit. In populations with restricted BMI ranges, BRI offers no advantage over WHtR.

An alternative approach is the allometric framework, combining BMI (weight normalized for height) and ABSI (WC normalized for weight and height). Krakauer and Krakauer [43] demonstrated that only ABSI and the hip index (HI) are statistically independent of BMI among proposed anthropometric indices, and that their risk estimates can be combined to optimize overall risk assessment. A prime advantage is that the odds ratios for BMI and ABSI can be combined into a single OR, which outperforms any single index for mortality prediction, with a superior Akaike information score. Future studies should consider adopting this approach to examine whether combined BMI and ABSI provide better predictive value for subclinical atherosclerosis than individual indices.

The inverse height–cIMT correlation (*r* = −0.106, *p* < 0.01) supports the rationale for WHtR over WC: WC alone may be biased by not accounting for height, possibly explaining why WC was the only index associated with cIMT in partially adjusted models. It also relates to ABSI, whose independence from height may prevent it from capturing this protective effect—potentially contributing to the inverse ABSI–cIMT association.

Geraci et al. [1]—the most directly comparable study—found that BMI and BRI independently predicted vascular injury in hypertensive adults, whereas ABSI was not associated with cIMT. This contrasts with our null findings, but several sample differences plausibly explain the divergence: their patients had established hypertension (we excluded it), a wider age range (higher, more variable cIMT), greater variance in adiposity indices (we restricted BMI to 18.5–35 kg/m^2^), and no exclusions for dyslipidaemia or diabetes (we excluded both)—removing individuals in whom the adiposity–cIMT association is strongest. Notably, ABSI was not independently associated with cIMT in either study. Costo‐Muriel et al. [44] similarly found that traditional and novel anthropometric indices had limited discriminatory ability for subclinical carotid atherosclerosis. Chang et al. [37], in 10,907 rural Chinese adults, found that BRI outperformed other indices for left ventricular hypertrophy (AUROCs 0.74 and 0.67), suggesting BRI may have value for other cardiovascular phenotypes, though this relates to cardiac rather than carotid structure.

Our finding that ABSI was not independently associated with cIMT is consistent with prior evidence from the same population and from young adults. Haghighatdoost et al. [12], using the same TLGS parent cohort, found that ABSI had the weakest correlations and lowest AUC for cardiovascular risk factors and metabolic syndrome among 9555 Iranian adults. Similarly, Amirabdollahian and Haghighatdoost [13], in 550 young British adults aged 18–25 years using nearly identical analytical methods, found that WHtR was the best predictor of cardiometabolic risk, whereas ABSI showed the weakest correlation. Wu et al. [45], in middle‐aged and older adults, similarly reported a non‐significant negative correlation between ABSI and carotid wall thickness, consistent with our observation. These findings suggest that ABSI's poor performance is not specific to our sample but reflects a more general limitation of the index in young and middle‐aged populations.

In our study, the inverse association between ABSI and cIMT observed in the study may be explained by ABSI's unique nature as a body shape index that is statistically independent of BMI, reflecting complex aspects of body composition and fat distribution rather than just fat quantity. In young, healthy adults, higher ABSI may correspond to variations in lean mass or body shape patterns less related to atherosclerotic burden. Additionally, ethnic, sex and height‐WC differences in the studied population may influence ABSI's predictive ability. Non‐linear relationships and residual confounding factors, such as fitness, could also contribute to this unexpected inverse correlation. Furthermore, measurement complexities and adjustment for covariates might affect the association, indicating that ABSI captures distinct underlying biological factors not fully aligned with cIMT progression. Overall, these mechanisms highlight the complexity of interpreting ABSI in relation to early vascular changes in diverse populations. The discrepancies might have several explanations, including ethnic differences, different sizes and characteristics of study samples.

The discordance between the significant ABSI group difference (*p* < 0.0001, Table 1), the null bivariate correlation (*r* = −0.049, Table 2), and the AUC below 0.5 (0.457) indicates directional inversion—higher ABSI associated with lower cIMT—rather than merely poor discrimination. The most likely explanation is confounding by sex: males have higher ABSI but lower cIMT, whereas females have lower ABSI but higher cIMT—a pattern consistent with Simpson's paradox. A non‐linear relationship or the narrow ABSI range may also contribute. Although the confidence interval includes 0.5, the point estimate supports the inverse direction, and clinically this does not support ABSI use for identifying elevated cIMT in young adults. Formal testing of effect modification by sex was not possible due to insufficient cases within each sex stratum (31 males, 58 females), so sex confounding remains the most plausible explanation but requires confirmation in larger studies.

The covariates in Model 4 may act as confounders or mediators. Under confounding, they are common causes of adiposity and cIMT, and adjustment removes spurious association. Under mediation, they lie on the causal pathway (adiposity → dyslipidaemia and elevated BP → atherosclerosis), and adjustment removes the indirect effect. Given the temporal precedence of adiposity, mediation is at least as plausible; the attenuation in Model 4 may therefore reflect removal of the mediated pathway rather than absence of association, making total effects (Models 1–3) potentially more relevant than the direct effect. Consistent with this interpretation, ROC analyses demonstrated that no anthropometric index—including WHtR and BRI, which yielded the numerically highest AUCs—achieved statistically significant discrimination of elevated cIMT, with all AUC values close to 0.5 and indistinguishable from chance.

This study has several strengths, including the use of a large, community‐based cohort from the TLGS, which provides robust and diverse population data. Comprehensive multivariable analyses were performed to adjust for various confounders, including demographic, metabolic and lifestyle factors. However, the cross‐sectional design limits causal inference between obesity indices and vascular changes. The study population consisted mainly of young, healthy adults, which may restrict generalisability to older or clinical populations. Moreover, potential residual confounding due to unmeasured factors such as muscle mass or nutritional status cannot be excluded. Because our study excluded participants with diabetes, our findings may not be generalisable to diabetic populations, in whom obesity indices have been associated with carotid plaque [46]. The study may be underpowered to detect modest AUC differences, and the observed AUCs were close to chance with wide confidence intervals; the null ROC findings should therefore be considered inconclusive rather than definitive, and larger studies are needed. Additionally, the absence of ECG gating—with cIMT varying by 0.04–0.09 mm across the cardiac cycle, comparable to the differences we aimed to detect—would attenuate associations toward the null and may partly explain the weak findings. Finally, ethnic and sex‐specific differences may influence the performance of novel indices like ABSI and BRI, necessitating caution when extrapolating findings to other populations. Future studies should also consider composite indices that combine ABSI with metabolic markers—such as TyG‐ABSI—which have shown significant associations with carotid plaque in other populations [47]. The associations observed in this study should therefore be interpreted with caution, and future studies with higher participation rates are needed to confirm these findings.

## 5 Conclusion

In conclusion, in young healthy adults, no anthropometric index—traditional or emerging—demonstrated statistically significant discriminative ability for elevated cIMT; all AUC values were close to chance, and the study was underpowered to detect modest differences. The null findings should be interpreted as inconclusive rather than definitive and do not support the clinical use of BRI, ABSI or WHtR for identifying subclinical atherosclerosis in this age group. Although BRI and WHtR showed modest correlations, WC was an independent predictor only in some models, and ABSI showed an inverse relationship—likely reflecting its unique characteristics and complex interaction with body composition and vascular health—none survived full adjustment; any association between adiposity and early subclinical atherosclerosis, if present, is likely small and undetectable with the current sample size. Future longitudinal studies with adequate power are needed to determine whether these indices have clinical utility in young adults.

## Author Contributions

**Behnaz Abiri:** conceptualization, writing – original draft, writing – review and editing, supervision. **Amirhossein Ramezani Ahmadi:** formal analysis. **Fereidoun Azizi:** writing – review and editing. **Pooneh Dehghan:** data curation. **Nika Farsinajad:** writing – review and editing. **Farhad Hosseinpanah:** conceptualization, writing – review and editing. **Maryam Mataji‐Amirroud:** writing – review and editing. **Azra Ramezankhani:** writing – review and editing. **Majid Valizadeh:** writing – review and editing.

## Funding

This study was conducted within the framework of the Tehran Lipid and Glucose Study (TLGS), which is supported by the Research Institute for Endocrine Sciences (RIES) of Shahid Beheshti University of Medical Sciences.

## Ethics Statement

This study was approved by the ethics committee of Research Institute for Endocrine Sciences (RIES) of Shahid Beheshti University of Medical Sciences (code: IR.SBMU.ENDOCRINE.REC.1403.041). All ethical principles outlined in the Declaration of Helsinki were strictly adhered to, and each participant provided written informed consent before enrollment.

## Consent

All authors have given consent for the paper to be published by the corresponding author.

## Conflicts of Interest

The authors declare no conflicts of interest.

## Data Availability

The data that supports the findings of this study are available on request from the corresponding authors, M.V.
